# Integrative analysis of the transcriptome, proteomics and metabolomics reveals key genes involved in the regulation of breast muscle metabolites in capons

**DOI:** 10.1186/s12864-024-11142-z

**Published:** 2024-12-23

**Authors:** Fei Ye, Zhi-dan Deng, Kun-yu Liu, Xiu-mei Yao, Wen-xiao Zheng, Qiong Yin, Xiang Hai, Jian-kang Gan, Zheng-Fen Zhang, Zheng Ma, Hua Li

**Affiliations:** 1 Guangdong Provincial Key Laboratory of Animal Molecular Design and Precise Breeding, Foshan University, Foshan, 528225 P.R. China; 2 Guangdong Tinoo’s Foods Co., Ltd, Qingyuan, Guangdong 511500 P.R. China

**Keywords:** Capons, Inostine-5'-monophosphate, Lipid metabolites, Metabolic pathways, GSTA2, Citric acid

## Abstract

Castration is widely used in poultry and livestock to enhance fat metabolism and improve the flavor, tenderness and juiciness of meat. However, the genetic regulatory mechanism underlying castration consequences have not been clarified. To investigate the key metabolites affecting the quality of capons and the key regulatory mechanisms, Qingyuan partridge roosters were subjected to castration. Metabolic profiling was used to detect differential metabolites in the breast muscle of both capon and control groups. Additionally, an integrative analysis of transcriptomics and proteomics was conducted to explore the genetic regulation mechanisms influencing meat quality. The results indicated that the muscle fiber density and shear force of capons was lower than that of normal chickens, and the fat percentage of capon group (CAM) was higher than control group (COM). The expression of the metabolite inostine-5’-monophosphate (IMP) was lower in capons, and lipid metabolites (PC (10:0/10:0), PC (6:0/13:1), LPC 22:6, LPC 18:2, LPE 18:1, LPE 20:4) were higher in capons. Metabolic pathways were found to be a common signaling pathway in all omics. *Glutamate-ammonia ligase (GLUL)*,* acetyl-CoA carboxylase beta (ACACB)*,* 1-acylglycerol-3-phosphate O-acyltransferase 2 (AGPAT2)*,* 4-hydroxy-2-oxoglutarate aldolase 1 (HOGA1)* and glutathione S-transferase alpha 2 (GSTA2) regulate the expression of citric acid, arachidonic acid, palmitic acid, isocitric acid, and betaine. These findings highlight the key mechanisms contributing to the meat quality differences between capons and normal chickens.

## Introduction

Capons are roosters that have undergone surgical removal of the testicles. In comparison to non-castrated roosters, capons exhibit a more docile temperament, reduced activity levels, enhanced feed conversion efficiency, and produce meat that is considered more flavorful and tender. The process of castration influences various meat characteristics, including color, muscle fiber type, overall composition, fat deposition, and other quality attributes [1–4]. Notably, capon meat is less fibrous than that of intact male chickens [5]. Furthermore, a high oxidative metabolism in muscle fibers can result in a dark red coloration of the flesh [6]. Surgical castration in roosters leads to consistent alterations in color parameters, characterized by increased L∗ and b∗ and lower a∗ in roosters [2, 7–10]. Additionally, the expression levels of genes associated with myofiber type, including *myosin*,* heavy chain 1B (MYH1B)*,* myostatin (MSTN)*, and *myoblast determination protein (MYOD)* genes, were modified, resulting in an enlarged cross-sectional area of muscle following caponization [11]. Furthermore, capons exhibited elevated levels of intramuscular fat, as well as higher concentrations of monounsaturated fatty acids and crude protein compared to normal chickens [12, 13].

Castration led to significant elevations in serum concentrations of high-density lipoprotein (HDL), low-density lipoprotein (LDL) and total cholesterol (TC) [14]. Additionally, castration resulted in an increased the monounsaturated fatty acid content and a high polyunsaturated fatty acid (PUFA) to saturated fatty acid (SFA) ratio. The predominant fatty acids identified were oleic acid (C18:1), palmitic acid (C16:0), and linoleic acid (C18:2). The contents of C18:1 in capons were higher than those in roosters, whereas the levels of butyric acid (C4:0), octanoic acid (C8:0), stearic acid (C18:0) and arachidonic acid (C20:4) were lower in capons than in roosters. Caponization was found to enhance adipocyte differentiation by upregulating the expression of *fatty acid synthase (FAS)*,* lipoprotein lipase (LPL)*, and *peroxisome proliferator-activated receptor γ (PPARγ)* in the liver [14]. The meat quality of capons surpasses that of normal chickens, attributed to differences in muscle fiber properties, muscle composition and intramuscular fat content. However, the molecular mechanisms underlying these differences remain uncharacterized. In this study, metabolomics was employed to investigate the composition of capon muscle, and the transcriptome and proteome were used to analyze the relevant regulatory mechanisms to provide a theoretical reference for meat quality improvement through muscle fiber development of capons.

## Results

### The differences in breast muscle between the capon group (CAM) and control group (COM)

**Fig. 1:**
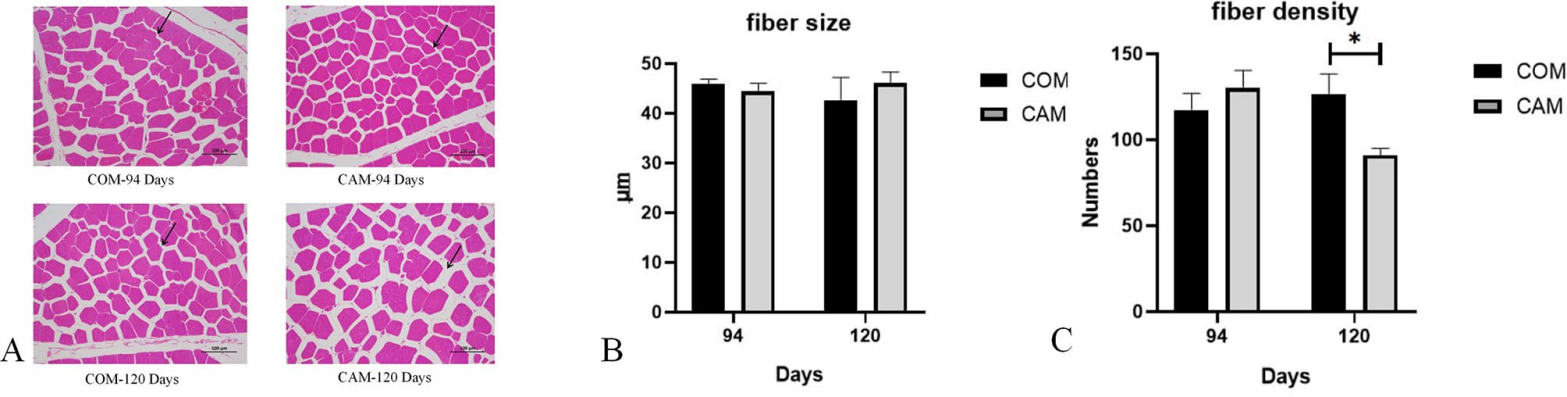
Comparison of muscle fibers between CAM and COM groups (200x, *n* = 3). **A**: HE stains of muscle; **B**: Fiber density of CAM and COM groups; **C**: Fiber size of CAM and COM groups. (*) indicates significance at *P* < 0.05

At 94 days, the Hematoxylin and Eosin (H&E) staining of muscle fibers (Fig. 1A-C) showed that there was no significant difference in either muscle fiber density or size between the CAM group and the COM group. Similarly, at 120 days, there was no significant difference in muscle fiber size between the two groups. However, the muscle fiber density in the CAM group was significantly lower than that in the COM group.

**Fig. 2:**
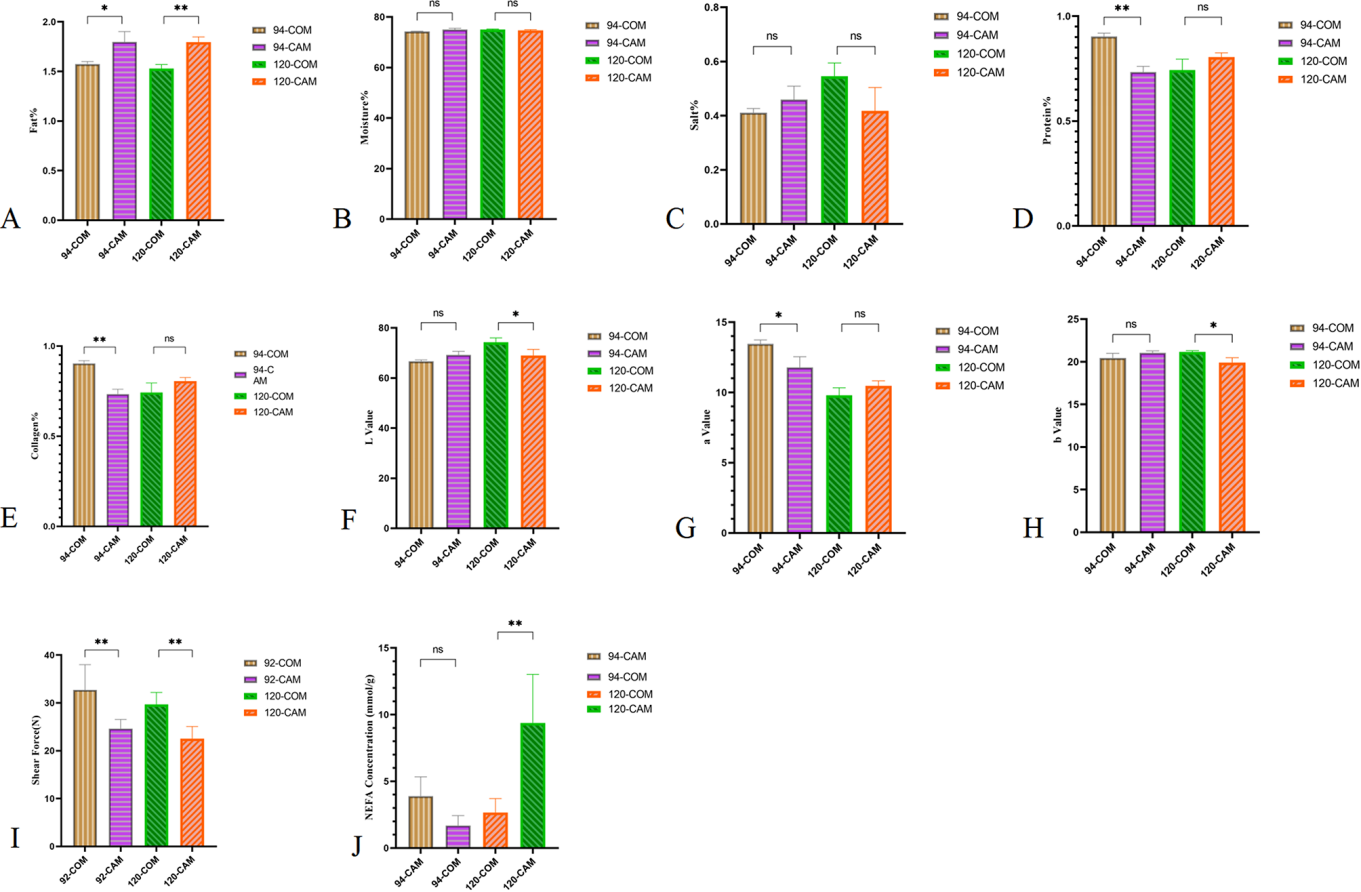
Comprehensive comparison of breast muscle attributes between CAM and COM groups (*n* = 3). **A**: Fat percentage of breast muscle; **B**: Moistute percentage of breast muscle; **C**: Salt percentage of breast muscle; **D**: Protein percentage of breast muscle; **E**: Collagen Protein percentage of breast muscle; **F**: L* value of breast muscle; **G**: a* value of breast muscle; **H**: b* value of breast muscle; **I**: Shear force of breast muscle; J: NEFA content of breast muscle. (*) indicates significance at *P* < 0.05. (**) indicates significance at *P* < 0.01. (ns) indicates significance at *P >* 0.05

The results of the meat composition analysis (Fig. 2A-H) showed the CAM group exhibited a significantly (*P* < 0.05) higher fat content at 94 and 120 days, and the fat percentage of CAM group was extremely significant (*P* < 0.001) higher than COM group at 120 days. Additionally, the shear force values were significantly (*P* < 0.001) greater in the COM group than those in the CAM group (Fig. 2I). In contrast, the non-esterified fatty acid (NEFA) assay revealed a marked difference between the two groups (Fig. 2J), with the COM group having significantly (*P* < 0.001) lower NEFA levels than the CAM group.

Consequently, the 120-day-old CAM group and COM group were selected for sequencing to investigate the genetic regulation underlying the differences between capons and normal chickens.

### Differentially expressed metabolites (DEMs) between the CAM and COM groups

**Fig. 3:**
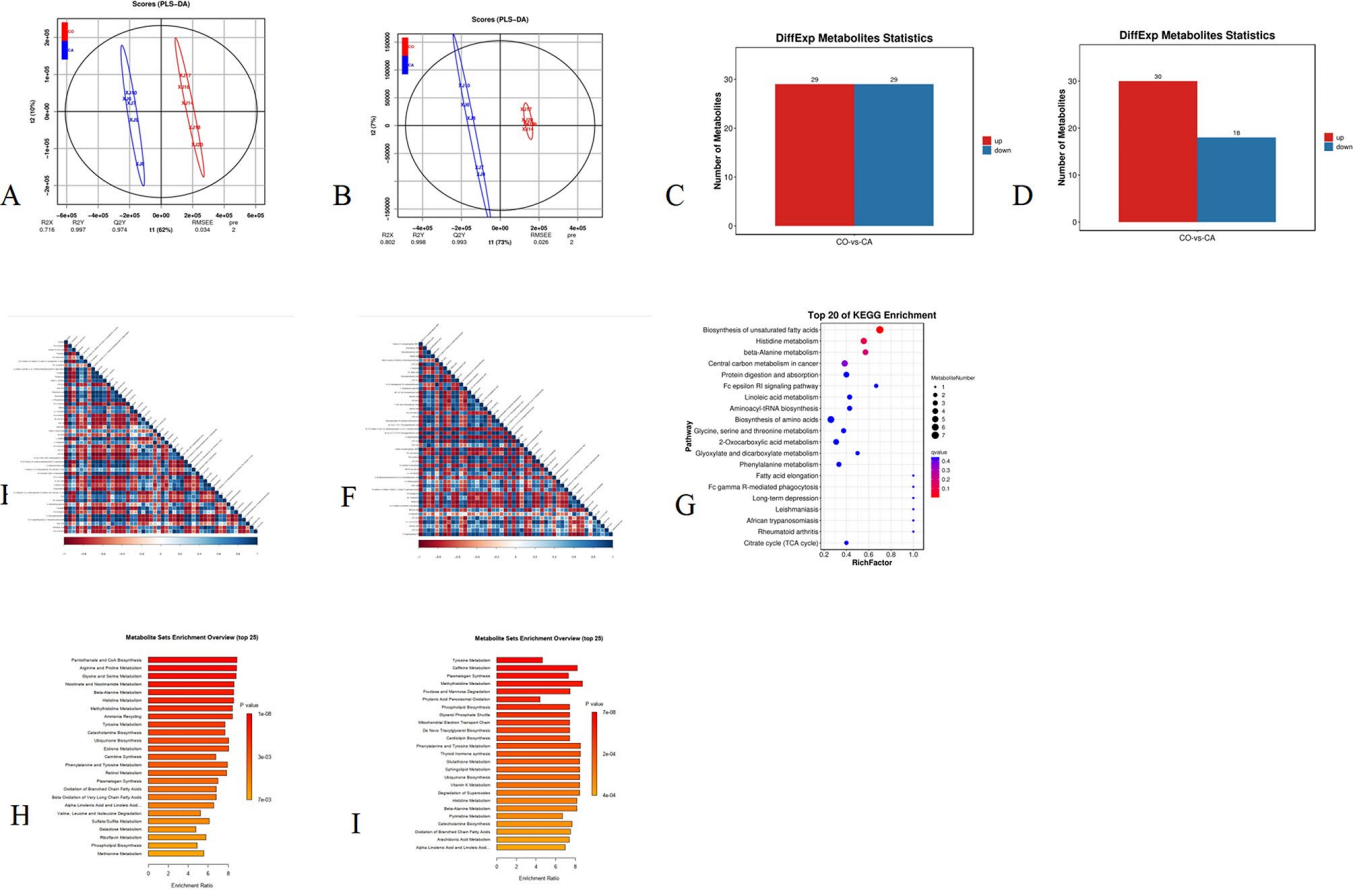
Analysis of DEMs of the COM and CAM groups (*n* = 5). **A**: The score of OPLS-DA of POS ion mode. **B**: The score of OPLS-DA of NEG ion mode. **C**: Statistics of DEMs of POS ion mode. **D**: Statistics of DEMs of NEG ion mode. **E**: Heat map of DEMs correlations of POS ion mode. **F**: Heat map of DEMs correlations of NEG ion mode. **G**: KEGG analysis of DEMs. **H**: Metabolite set enrichment analysis of POS ion mode. I: Metabolite set enrichment analysis of NEG ion mode

The orthogonal least partial squares discriminant analysis (OPLS-DA) score was 62% for positive (POS) ion mode (Fig. 3A) and 73% for negative (NEG) ion mode (Fig. 3B), and the sequencing results could be used for further analysis. A total of 29 upregulated and 29 downregulated metabolites were detected in POS ion mode (Fig. 3C). These metabolites were involved in the class of carboxylic acids and derivatives, cinnamic acids and derivatives, fatty acyls, and steroids and steroid derivatives (Table 1 and Supplementary file 1). A total of 30 upregulated metabolites and 18 downregulated metabolites were detected by NEG ion mode (Fig. 3D). These metabolites were involved in the classes of hydroxy acids and derivatives, carboxylic acids and derivatives, indoles and derivatives, fatty acyls, benzene and benzene close derivatives (Table 2 and Supplementary file 1). In POS mode, there were positive correlations among L-histidine, creatinine, 2-hydroxyphenylalanine, L-phenylalanine, creatine and betaine of the carboxylic acid and derivative class (Fig. 3E). In the NEG mode, there were positive correlations between palmitic acid, elaidic acid, adrenic acid, 8Z,11Z, 14Z-eicosatrienoic acid, docosapentaenoic acid and arachidonic acid of the fatty acyls (Fig. 3F). Kyoto Encyclopedia of Genes and Genomes (KEGG) results showed that the metabolites were enriched in the signaling pathways of biosynthesis of unsaturated fatty acids, histidine metabolism and biosynthesis of amino acids (Fig. 3G and Supplementary file 2). Metabolite set enrichment analysis (MSEA) showed that the DEMs of the POS mode were enriched in pantothenate and CoA biosynthesis, arginine and proline metabolism, and glycine and serine metabolism (Fig. 3H and Supplementary file 3). The DEMs of the NEG mode were enriched in methylhistidine metabolism, caffeine metabolism, and glutathione metabolism (Fig. 3I and Supplementary file 4).

**Table 1:** The part DEMs of POS ion mode

index	log2_FC (case_mean/control_mean)	Pvalue	fdr	vip	name	formula	Class
**Com_111_pos**	-2.984856578	1.43E-08	2.12E-06	2.77617902	Pantothenic acid	C9 H17 N O5	Alcohols and polyols
**Com_625_pos**	3.806001199	0.00015802	0.001257362	1.067056951	Pyrogallol	C6 H6 O3	Benzene and substituted derivatives
**Com_105_pos**	-2.888222366	5.86E-05	0.00060232	2.736153135	L-Phenylalanine	C9 H11 N O2	Carboxylic acids and derivatives
**Com_250_pos**	-5.596459651	4.56E-06	0.000107325	1.806954132	L-Histidine	C6 H9 N3 O2	Carboxylic acids and derivatives
**Com_519_pos**	-2.990773108	1.68E-07	1.13E-05	1.034760078	2-Hydroxyphenylalanine	C9 H11 N O3	Carboxylic acids and derivatives
**Com_6_pos**	-2.014401148	3.88E-08	3.19E-06	13.07714876	Creatine	C4 H9 N3 O2	Carboxylic acids and derivatives
**Com_74_pos**	-3.031349139	8.69E-06	0.000160224	3.395946642	Creatinine	C4 H7 N3 O	Carboxylic acids and derivatives
**Com_76_pos**	-1.254682781	0.006770065	0.019121557	2.514990772	Betaine	C5 H11 N O2	Carboxylic acids and derivatives
**Com_299_pos**	-4.21477454	7.76E-06	0.000151138	1.517532937	2-Hydroxycinnamic acid	C9 H8 O3	Cinnamic acids and derivatives
**Com_388_pos**	2.816723464	0.00216382	0.007810864	1.235258548	9-Oxo-ODE	C18 H30 O3	Fatty Acyls
**Com_527_pos**	3.598082809	0.001547372	0.006202533	1.014459081	Palmitoleic Acid	C16 H30 O2	Fatty Acyls
**Com_61_pos**	-2.291045857	0.001028099	0.004754956	3.266545543	Acetyl-L-carnitine	C9 H17 N O4	Fatty Acyls
**Com_151_pos**	1.709125901	0.010744033	0.026990575	1.8108324	Monoolein	C21 H40 O4	Glycerolipids
**Com_665_pos**	4.330236676	1.33E-05	0.000209347	1.011431079	1-Oleoyl-Sn-Glycero-3-Phosphocholine	C26 H52 N O7 P	Glycerophospholipids
**Com_117_pos**	-1.522318858	0.030974491	0.057590762	1.775526842	L-Tryptophan	C11 H12 N2 O2	Indoles and derivatives
**Com_130_pos**	-1.954458713	0.041819025	0.073506125	1.477600358	7-hydroxy-3-(4-methoxyphenyl)-4 H-chromen-4-one	C16 H12 O4	Isoflavonoids
**Com_13_pos**	-2.906128353	1.64E-06	4.67E-05	7.400751472	Carnosine	C9 H14 N4 O3	Peptidomimetics
**Com_112_pos**	-2.198425939	1.12E-06	4.05E-05	2.765438249	Nicotinamide	C6 H6 N2 O	Pyridines and derivatives
**Com_234_pos**	-2.137593322	3.07E-09	1.14E-06	1.368359963	5-Methyl-2’-deoxycytidine	C10 H15 N3 O4	Pyrimidine nucleosides
**Com_392_pos**	1.597190723	6.68E-06	0.000137343	1.352863305	7-Ketolithocholic acid	C24 H38 O4	Steroids and steroid derivatives
**Com_515_pos**	4.323030274	0.000614363	0.003404573	1.181245756	Cholecalciferol	C27 H44 O	Steroids and steroid derivatives

**Table 2:** The part DEMs of NEG ion mode

index	log2_FC (case_mean /control_mean)	Pvalue	fdr	vip	name	formula	Class
**Com_263_neg**	3.764408615	0.002418218	0.010382217	1.078647075	Benzoic acid	C7 H6 O2	Benzene and substituted derivatives
**Com_141_neg**	-7.51644417	3.85E-07	2.48E-05	2.093609219	3-Methylhistidine	C7 H11 N3 O2	Carboxylic acids and derivatives
**Com_294_neg**	-5.76908727	0.000642659	0.003941643	1.178172591	2-Furoylglycine	C7 H7 N O4	Carboxylic acids and derivatives
**Com_93_neg**	1.758319602	0.003171815	0.012221324	1.196282842	Citric acid	C6 H8 O7	Carboxylic acids and derivatives
**Com_95_neg**	1.604125868	0.042063089	0.07166304	1.045639459	Isocitric acid	C6 H8 O7	Carboxylic acids and derivatives
**Com_1_neg**	1.94706518	0.000304049	0.002365584	10.18663214	Arachidonic acid	C20 H32 O2	Fatty Acyls
**Com_14_neg**	1.049241857	0.000173675	0.001553424	4.840091711	Elaidic acid	C18 H34 O2	Fatty Acyls
**Com_27_neg**	1.704048171	0.00159933	0.007686333	3.905852042	Docosapentaenoic acid	C22 H34 O2	Fatty Acyls
**Com_42_neg**	1.450153746	0.000578503	0.003840779	3.240565657	8Z,11Z,14Z-Eicosatrienoic acid	C20 H34 O2	Fatty Acyls
**Com_45_neg**	1.014180168	0.000409897	0.002965999	2.997129467	Palmitic acid	C16 H32 O2	Fatty Acyls
**Com_47_neg**	1.216695754	0.00403729	0.014474571	2.678471862	Adrenic acid	C22 H36 O2	Fatty Acyls
**Com_55_neg**	1.984002546	0.003792997	0.013958229	2.518235605	cis-5,8,11,14,17-Eicosapentaenoic acid	C20 H30 O2	Fatty Acyls
**Com_7_neg**	2.470298406	0.01384229	0.033895189	5.533575826	Docosahexaenoic acid	C22 H32 O2	Fatty Acyls
**Com_36_neg**	-5.827957286	0.002968311	0.011799951	4.147288986	DL-Malic acid	C4 H6 O5	Hydroxy acids and derivatives
**Com_268_neg**	-3.127725269	0.000638201	0.003941643	1.165716833	DL-Tryptophan	C11 H12 N2 O2	Indoles and derivatives
**Com_476_neg**	-7.773616186	3.58E-07	2.48E-05	1.139964275	Histamine	C5 H9 N3	Organonitrogen compounds
**Com_160_neg**	-6.158463065	0.000286454	0.002277482	1.531502007	D-Xylulose 5-phosphate	C5 H11 O8 P	Organooxygen compounds
**Com_46_neg**	-5.171510216	2.88E-05	0.000431013	4.151194353	L-Anserine	C10 H16 N4 O3	Peptidomimetics

### Differentially expressed genes (DEGs) of the CAM and COM groups

**Fig. 4:**
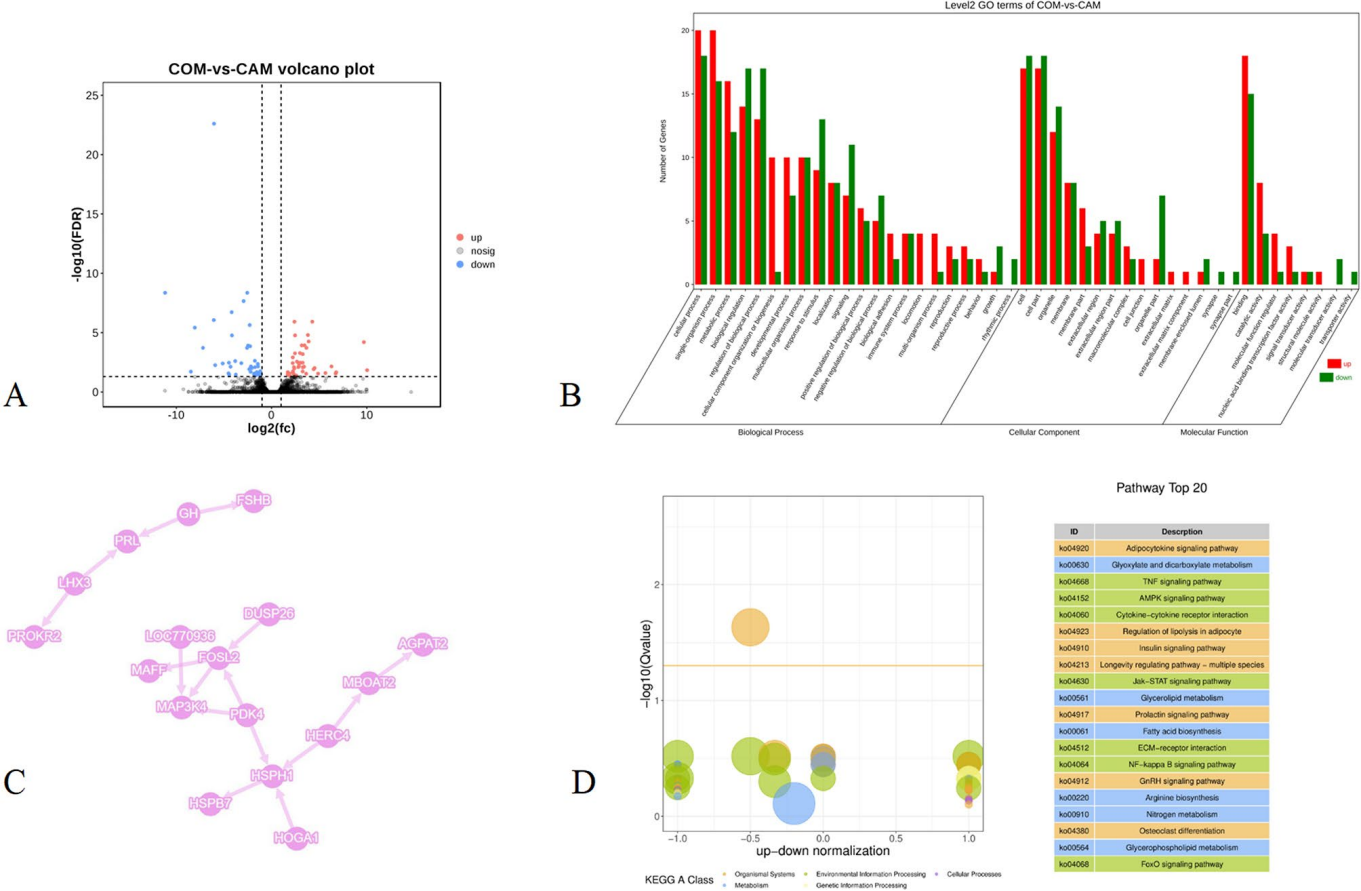
Analysis of DEGs of the COM and CAM groups (*n* = 3). **A**: Statistics of deferentially expressed genes. **B**: GO enrichment of DEGs. **C**: KEGG enrichment of DEGs. D: String interaction network of DEGs

The RNA-seq results showed that compared with the capon group, 44 genes were upregulated, including *4-hydroxy-2-oxoglutarate aldolase mitochondrial (HOGA1)*, g*uanylate-binding protein 7 Isoform X1 (GBP7)*, and *cysteine and glycine-rich protein 3 (CSRP3)*, while 43 genes were downregulated, including *follitropin subunit beta precursor (FSHB)*, *LIM/homeobox protein Lhx3 ISOform X1 (Lhx3)*, and *gastrin-releasing peptide precursor (GRP)* (Fig. 4A and Supplementary file 5). These DEGs formed an interaction network with *heat shock protein 105 kDa (HSPH1)*,* prolactin precursor (PRL)*,* pyruvate dehydrogenase kinase*,* isozyme 4 (PDK4)*, and *andfos-related antigen 2 (FOSL2)* as the core (Fig. 4D). DEGs were enriched in the adipocytokine signaling pathway, glyoxylate and dicarboxylate metabolism, glycerolipid metabolism, glycerolipid metabolism, and fatty acid biosynthesis (Fig. 4C and Supplementary file 6) and participated in receptor binding, G-protein coupled receptor binding, and other functions (Fig. 4B and Supplementary file 7).

### Differentially expressed proteins (DEPs) of the CAM and COM groups

**Fig. 5:**
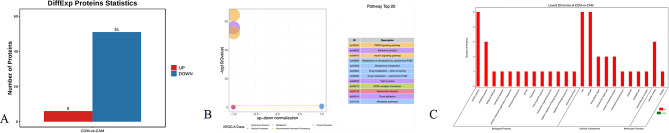
Analysis of DEPs of the COM and CAM groups (*n* = 3). **A**: Statistics of differentially expressed proteins. **B**: GO enrichment of DEPs. **C**: KEGG enrichment of DEPs

Data independent acquisition (DIA) proteomics detected 6 proteins (glutathione S-transferase alpha 2, transcript variant X2 (GSTA2), albumin (ALB), collagen type VI alpha 1 chain (COL6A1)) that were upregulated and 51 downregulated proteins, including myosin heavy chain 1 F (MYH1F), sorbin and SH3 domain containing 1, transcript Variant X1 (SORBS1), and heat shock protein Family B member 2 (HSPB2) (Fig. 5A and Supplementary file 8). These proteins were enriched in the peroxisome proliferator-activated receptor (PPAR) signaling pathway, adherens junction, insulin signaling pathway, glutathione metabolism, and other signaling pathways (Fig. 5C and Supplementary file 9) and were involved in glutathione metabolic process, fatty acid binding, and glutathione metabolic process (Fig. 5B and Supplementary file 10).

### Combined analysis of the transcriptome and metabolome

Combined transcriptome and metabolome analysis showed that there were 17 pathways enriched in both DEGs and DEMs. The *P* values of glyoxylate and dicarboxylate metabolism and regulation of lipolysis in adipocytes were significant (Table 3).

**Table 3:** The common pathways of transcriptome and metabolome

#	Pathway	Candidate genes with pathway annotation (31)	Gene_Pvalue	Gene_Qvalue	Metabolite_CO-vs-CA (31)	Metabolite_Pvalue	Metabolite_Qvalue	Pathway ID
**1**	Glyoxylate and dicarboxylate metabolism	2 (6.45%)	0.010776	0.303023	2	0.094702	0.44135	ko00630
**2**	Regulation of lipolysis in adipocyte	2 (6.45%)	0.027734	0.303023	1	0.532071	0.628812	ko04923
**3**	Fatty acid biosynthesis	1 (3.23%)	0.064674	0.356908	2	0.197836	0.455252	ko00061
**4**	GnRH signaling pathway	2 (6.45%)	0.075605	0.356908	1	0.260324	0.455252	ko04912
**5**	Alanine, aspartate and glutamate metabolism	1 (3.23%)	0.156339	0.470471	1	0.4545	0.602908	ko00250
**6**	Neuroactive ligand-receptor interaction	3 (9.68%)	0.191903	0.470471	1	0.656481	0.711188	ko04080
**7**	Arginine and proline metabolism	1 (3.23%)	0.198849	0.472267	3	0.14938	0.44135	ko00330
**8**	Ovarian Steroidogenesis	1 (3.23%)	0.227365	0.502377	1	0.656481	0.711188	ko04913
**9**	Taste transduction	1 (3.23%)	0.254897	0.509794	1	0.656481	0.711188	ko04742
**10**	Biosynthesis of amino acids	1 (3.23%)	0.277739	0.541234	6	0.079453	0.44135	ko01230
**11**	Long-term depression	1 (3.23%)	0.285201	0.541881	1	0.13964	0.44135	ko04730
**12**	Aldosterone synthesis and secretion	1 (3.23%)	0.338836	0.569569	1	0.816599	0.842523	ko04925
**13**	Glucagon signaling pathway	1 (3.23%)	0.378921	0.569569	2	0.253542	0.455252	ko04922
**14**	Serotonergic synapse	1 (3.23%)	0.413591	0.569569	2	0.419298	0.602908	ko04726
**15**	Vascular smooth muscle contraction	1 (3.23%)	0.474652	0.588553	1	0.364551	0.551065	ko04270
**16**	Platelet activation	1 (3.23%)	0.480136	0.588553	1	0.4545	0.602908	ko04611
**17**	Metabolic pathways	5 (16.13%)	0.76661	0.776831	19	0.844203	0.857394	ko01100

**Fig. 6:**
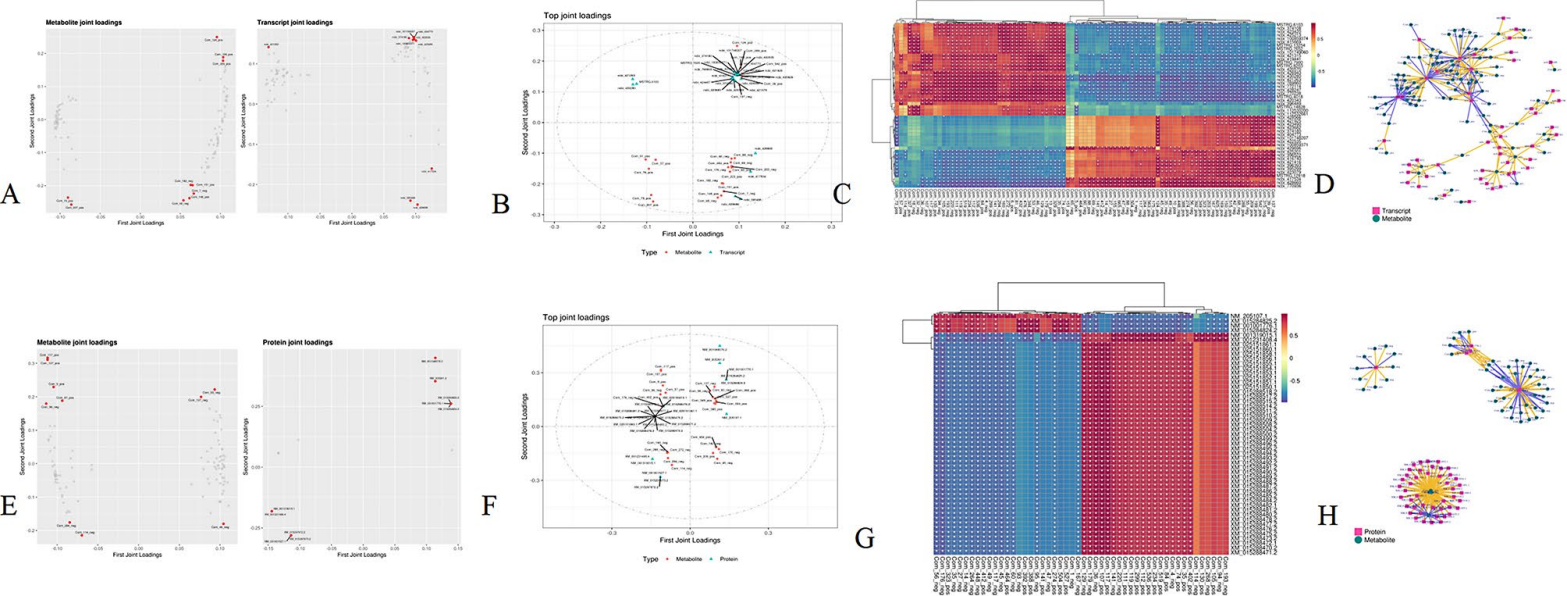
O2PLS loading plot. Positive or negative loading value indicate positive or negative correlations with another group. The larger the absolute value of the loading, the stronger the association. **A**: Loading map of metabolome and transcriptome; **B**: Squares of loading values of the first two dimensions and the top 25 genes and metabolites were integrated to draw loading maps. **C**: Heat map of correlation between gene expression and metabolite. **D**: Network map of correlation between gene expression and metabolite. **E**: Loading map of metabolome and transcriptome; **F**: Squares of loading values of the first two dimensions and the top 25 genes and metabolites were integrated to draw loading maps. **G**: Heat map of correlation between protein expression and metabolite. **H**: Network map of correlation between protein expression and metabolite

Loading plot analysis of 3 components (Table 4) showed that metabolites LysoPC 18:3, PC (6:0/13:1) and LPE 18:1 were positively correlated with loading 1 and loading 2; LPS 18:0, docosahexaenoic acid, LPC 18:0, monoolein and LPC 22:6 were positively correlated with loading 1 and negatively correlated with loading 2; L(-)-carnitine and ACar 18:0 were negatively correlated with loading 1 and loading 2 (Fig. 6A and Supplementary file 11). *Prokineticin receptor 1 isoform X1 (PROKR2)* was negatively correlated with loading 1 and positively correlated with loading 2; *macrophage receptor (MARCO)*,* GBP7* and *C-C motif chemokine 3-like 1 (CCL26)* were positively correlated with loading 1 and negatively correlated with loading 2; *musculoskeletal embryonic nuclear protein 1 (MUSTN1)*,* protein enabled homolog isoform X1 (ENAH)*,* nephronectin isoform X1 (NPNT)*,* proteoglycan 4 (PRG4)* and *nutritionally regulated adipose and cardiac enriched protein homolog isoform X1 (C14orf180)* were positively correlated with loading 1 and loading 2 (Fig. 6A and Supplementary file 12).

**Table 4:** The common pathways of proteomic and metabolome

#	Pathway	Pathway ID	KEGG ID	differentially expressed genes or metabolites	description
**1**	Metabolic pathways	ko01100	C00078	Com_117_pos(L-Tryptophan)	(S)-alpha-Amino-beta-(3-indolyl)-propionic acid
			C00079	Com_105_pos(L-Phenylalanine)	(S)-alpha-Amino-beta-phenylpropionic acid
			C00135	Com_250_pos(L-Histidine)	(S)-alpha-Amino-1 H-imidazole-4-propionic acid
			C00153	Com_112_pos(Nicotinamide)	Vitamin PP
			C00158	Com_93_neg(Citric acid)	2-Hydroxytricarballylic acid
			C00180	Com_263_neg(Benzoic acid)	Dracylic acid
			C00219	Com_1_neg(Arachidonic acid)	cis-5,8,11,14-Eicosatetraenoic acid
			C00231	Com_160_neg(D-Xylulose 5-phosphate)	D-Xylulose 5-phosphate
			C00249	Com_45_neg(Palmitic acid)	Cetylic acid
			C00300	Com_6_pos(Creatine)	Methylglycocyamine
			C00311	Com_95_neg(Isocitric acid)	1-Hydroxypropane-1,2,3-tricarboxylic acid
			C00386	Com_13_pos(Carnosine)	Nalpha-(beta-alanyl)-L-histidine
			C00388	Com_476_neg(Histamine)	2-(4-Imidazolyl)ethylamine
			C00719	Com_76_pos(Betaine)	Trimethylammonioacetate
			C00791	Com_74_pos(Creatinine)	1-Methylglycocyamidine
			C00864	Com_111_pos(Pantothenic acid)	(R)-Pantothenate
			C01262	Com_46_neg(L-Anserine)	Anserine
			C03242	Com_42_neg(8Z,11Z,14Z-Eicosatrienoic acid)	8,11,14-Icosatrienoate
			C05443	Com_515_pos(Cholecalciferol)	Calciol
			K00799	XM_015284824.2(GSTA2)	glutathione S-transferase
			K00799	NM_001001776.1(GSTA2)	glutathione S-transferase
			K00799	XM_015284825.2(GSTA2)	glutathione S-transferase

The top 25 joint loadings showed the most associated genes and metabolites (Fig. 6B). *PROKR2* and *HSPB7* genes were positively correlated with loading 2 and negatively correlated with loading 1; acylcarnitine (ACar) 18:0, L(-)-carnitine, acetyl-L-carnitine, betaine and other metabolites were negatively correlated with loading 1 and loading 2; *interferon-induced very large GTPase 1 (GVINP1)*,* CCL26*, *MARCO* genes and LPE 20:4, glycerophospho-N-palmitoyl ethanolamine, lysophosphatidylethanolamine

(LPE) 18:0 metabolites were positively correlated with loading 1 and negatively correlated with loading 2; *MUSTN1*,* C14orf180*,* membrane-bound O-acyltransferase domain-containing protein 2 (MBOAT2)*,* serine/threonine-protein kinase SBK2-like (SBK2)* genes and lysophosphatidlycholine (LysoPC) 18:3, LPE 18:1, and phosphatidylcholine (PC) (10:0/10:0) metabolites were positively correlated with loading 1 and loading 2 (Fig. 6B).

The heatmap and network diagram of the top 250 DEMs and DEGs with Pearson’s coefficients are shown in Fig. 6C and D and Supplementary file 13. *GVINP1*,* 1-acyl-sn-glycerol-3-phosphate acyltransferase beta isoform X1 (AGPAT2)*,* F-box only protein 32 (FBXO32)*,* krueppel-like factor 9 (KLF9)*,* SRSF protein kinase 3-like (LOC770936)*, and *5-hydroxytryptamine receptor 3 A (HTR3A)* were core genes. Related metabolites included 9-Oxo-10(E), 12(E)-octadecadienoic acid, 5-methyl-2’-deoxycytidine, 3-methylhistidine, inosine-5’-monophosphate (IMP), and lysophosphatidylcholine (LPC) 20:4.

### Combined proteomic and metabolomic analyses

Metabolic pathways were the pathways enriched by DEPs and DEMs, with GSTA2 protein and L-histidine and anserine metabolites (Table 5). The bidirectional orthogonal projection to latent structures (O2PLS) analysis found 4 two-component associations (Table 4). Loading plot analysis of the four components showed that citric acid and 1,4-dithioerythritol were positively correlated with loading 1 and loading 2; palmitic acid was positively correlated with loading 1 and negatively correlated with loading 2; 2-furoylglycine and 4-oxoproline were negatively correlated with loading 1 and loading 2; and L-tryptophan, indole-3-acrylic acid, PC (18:2e/18:3), ACar 18:2, and DL-malic acid were positively correlated with loading 1 and negatively correlated with loading 2 (Fig. 6E and Supplementary file 14). The proteins vimentin (VIM), ALB and GSTA2 were positively correlated with loading 1 and loading 2; MYH1F and HSPB2 were negatively correlated with loading 1 and loading 2 (Fig. 6E and Supplementary file 15).

**Table 5:** O2PLS model parameters and prediction error values

#	Analysis	Model	nx	ny	*n*	Prediction error
1	**transcriptome and metabolome**	COM-vs-CAM	0	0	3	1.154998
2	**proteomic and metabolome**	COM-vs-CAM	0	0	4	1.189366

Note model: model of each comparison group; nx: number of components in the orthogonal part of the transcriptome; ny: number of components in the orthogonal part of the metabolome; n: the number of components of the two omics association; prediction error: prediction error of the mode

The top 25 joint loadings showed the proteins and metabolites with the highest degree of association. ALB, VIM, GSTA2 and COL6A1 proteins and palmitoleic acid, PC (14:1e/2:0), 9-Oxo-ODE, 2-arachidonoyl glycerol, and 1,4-dithioerythritol metabolites were positively correlated with loading 1 and loading 2; MYH1F, HSPB2 proteins and D-xylulose 5-phosphate, DL-tryptophan, 2-furoylglycine, 3-methylhistidine metabolites were negatively correlated with loading 1 and loading 2; SORBS1 and synaptopodin, transcript variant X3 (SYNPO) protein and L-tryptophan, indole-3-acrylic acid, PC (18:2e/18:3) metabolites were negatively correlated with loading 1 and positively correlated with loading 2; LPE 18:1, PC (18:2e/2:0), LPC 22:5, and palmitic acid metabolites were negatively correlated with loading 2 and positively correlated with loading 1 (Fig. 6F).

The heatmap and network diagram of the top 250 DEMs and DEPs with Pearson’s coefficients are shown in Fig. 6G and H and Supplementary file 16. GSTA2, COL6A1, and MYH1F were core proteins. Indole-3-acrylic acid, DL-malic acid, L-tryptophan, and LPC 22:5 were core metabolites.

### Combined analysis of the transcriptome and proteome

**Fig. 7:**
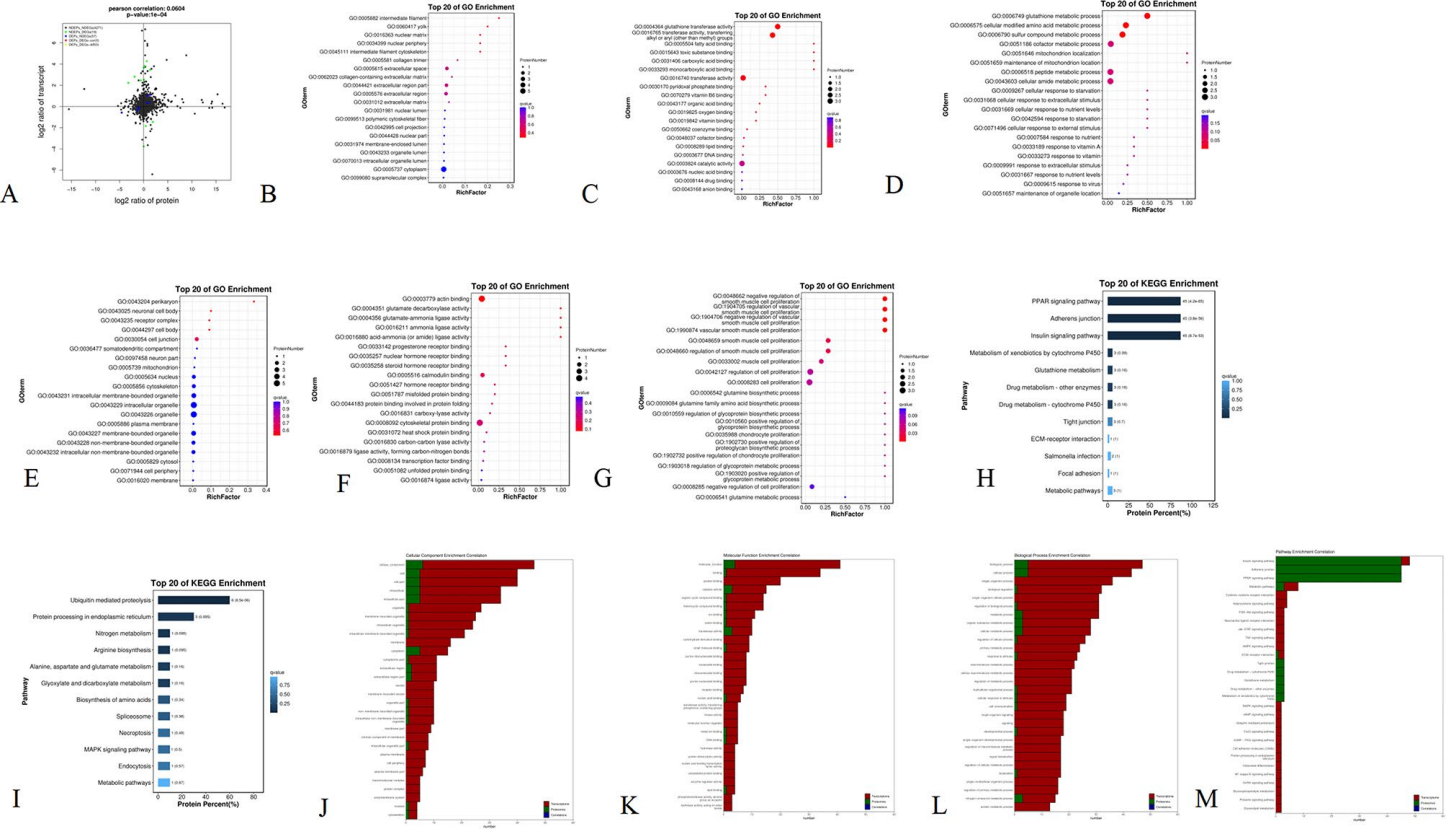
Conjoint analysis of the transcriptome and proteome. **A**: Four quadrants of DEGs and DEPs (the abscissa is the multiple of protein difference (log2); the ordinate is the multiple of transcriptome difference (log2), and the top of the figure shows the correlation coefficient and *P* value of the association between transcriptome and proteome). **B**-**G**: GO enrichments (**B**: DEPs-NDEGs (cellular component); **C**: DEPs-NDEGs (molecular function); **D**: DEPs-NDEGs (biological process); **E**: DEGs-NDEPs (cellular component); **F**: DEGs-NDEPs (molecular function); **G**: DEGs-NDEPs (biological process)). **H**-**I**: KEGG enrichments (**H**: DEPs-NDEGs; **I**: DEGs-NDEPs). **K**-**L**: Number of annotations of DEGs (red), DEPs (green), and common associated genes (blue) in GO term (**J**: DEGs-NDEPs (cellular component); **K**: DEGs-NDEPs (molecular function); **L**: DEGs-NDEPs (biological process)). **M**: Number of DEGs (red), DEPs (green), and co-associated genes (blue) annotated in the pathway

A total of 57 DEPs-nonDEGs (DEPs-NDEGs) and 19 DEGs-nonDEPs (DEGs-NDEPs) were found by combined transcriptomic and proteomic analysis, and no genes were associated with differences in the two omics (Fig. 7A and Supplementary file 17).

DEPs-NDEGs were enriched in the PPAR signaling pathway, adherens junction, insulin signaling pathway, and metabolism pathways (Fig. 7H and Supplementary file 18) and were involved in glutathione metabolic process, cellular modified amino acid metabolic process, cofactor metabolic process (biological process), cytoplasm, extracellular region, extracellular space (cellular component), catalytic activity, transferase activity, glutathione transferase activity, and fatty acid binding (molecular function) (Fig. 7B-D and Supplementary file 19).

DEGs-NDEPs were enriched in ubiquitin-mediated proteolysis, protein processing in endoplasmic reticulum, and metabolic pathways (Fig. 7I and Supplementary file 20) and were involved in cell proliferation, regulation of smooth muscle cell proliferation, muscle cell proliferation (biological process), organelle, cell junction (cellular component), actin binding, calmodulin binding, and cytoskeletal protein binding (molecular function) (Fig. 7E-G).

The gene ontology (GO) items commonly associated with the two omics analyses included cell, cytoplasm, intracellular (cellular component), catalytic activity, transferase activity, binding (molecular function), cellular process, metabolic process, and nitrogen compound metabolic process (biological process) (Fig. 7J-L and Supplementary file 21). The pathways commonly associated with the two omics analyses included the insulin signaling pathway, metabolic pathways and extracellular matrix-receptor (ECM-receptor) interaction (Fig. 7M).

### The relative expression of genes

**Fig. 8:**
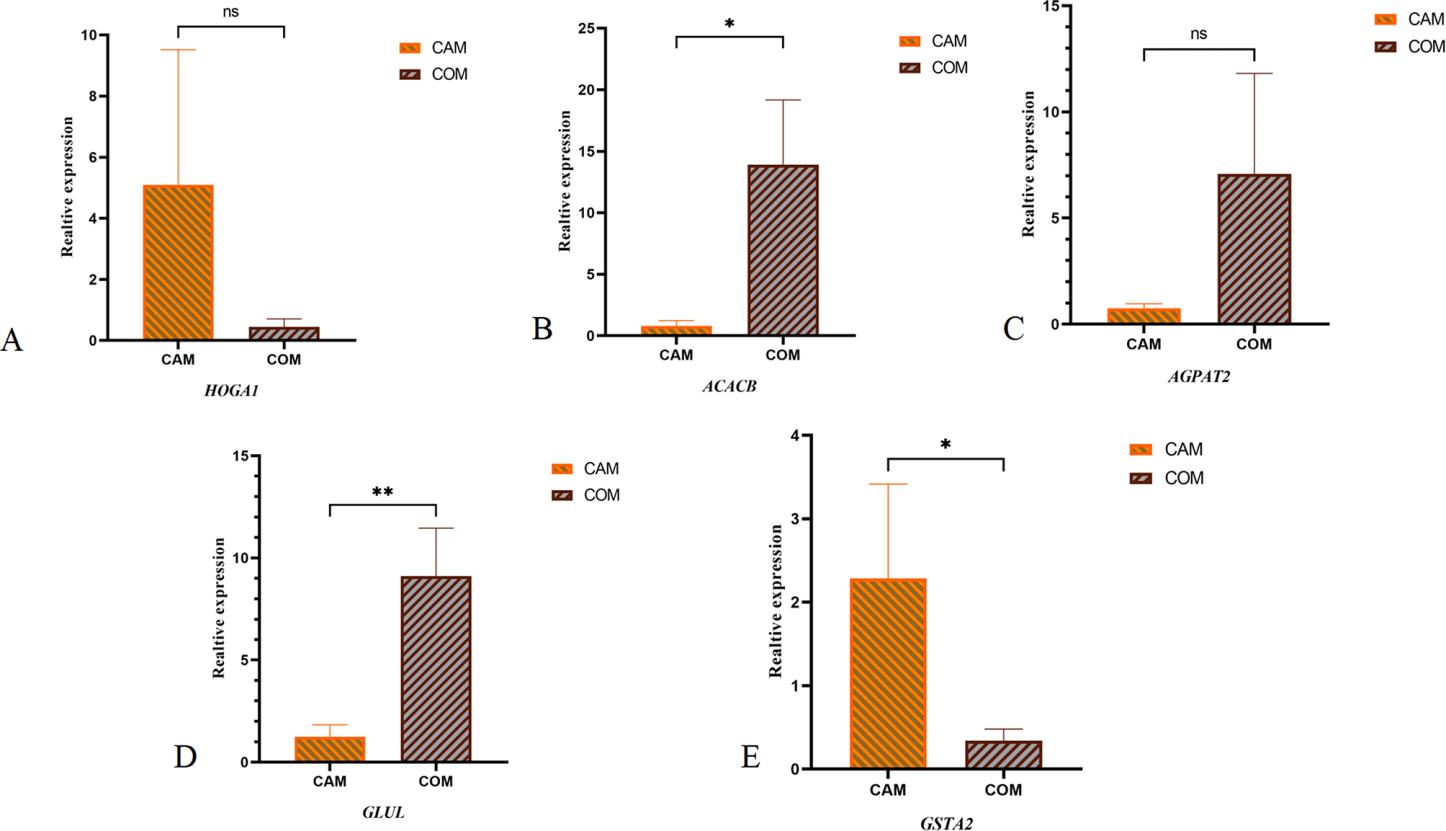
The relative expression of *HOGA1* (**A**), *ACACB* (**B**), *AGPAT2* (**C**), *GLUL* (**D**), and *GSTA2* (**E**). (*) indicates significance at *P* < 0.05. (**) indicates significance at *P* < 0.01. (ns) indicates significance at *P >* 0.05

Based on the aforementioned results, we identified five genes—*ACACB*,* GSTA2*,* HOGA1*,* GLUL*, and *AGPAT2*—as reliable key genes. The relative expression levels of *ACACB* (Fig. 8B) and *GLUL* (Fig. 8D), and *GSTA2* (Fig. 8E) exhibited significant (*P* < 0.05) differences between the CAM and COM groups at 120 days. In contrast, the relative expression analyses of *HOGA1* (Fig. 8A) and *AGPAT2* (Fig. 8C) revealed no significant differences.

## Discussion

Understanding the metabolism and development of breast muscle is crucial for improving meat quality, as it is the primary source of protein in broilers. The pectoral muscles consist of muscle fibers, connective tissue, and adipose tissue. In this study, after castrating 62-day-old Qingyuan partridge roosters, there was no significant change in muscle fiber diameter at ages 92 and 120 days compared to normal male chickens. This finding is inconsistent with the results of previous studies on muscle fiber diameter [15], which may be attributed to differences in chicken breeds and growth rates. However, the number of muscle fibers in 120-day-old capons was significantly fewer than normal, and the shear force values in the CAM group were significantly lower than those in the COM group, likely due to an increase in muscle fiber spacing. Consequently, muscle tenderness increased, leading to better meat quality in capons.

Moreover, significant differences were observed in the fat and protein content between the two groups. The CAM group exhibited higher fat content, while the COM group showed notably higher protein content. These compositional differences suggest that castration promotes fat deposition, which likely contributes to the enhanced juiciness and flavor of the capon meat.Castration increased the contents of myoglobin and monounsaturated fatty acids, with the main fatty acids were oleic acid (C18:1), palmitic acid (C16:0) and linoleic acid (C18:2), among which C18:1 had the highest content in capon muscle [12]. In this study, the lipid metabolites phosphatidylcholine (PC (10:0/10:0), PC (6:0/13:1), PC (14:1E/2:0), PC (18:2E/2:0)), lysophosphatidylcholine (LPC 22:6, LPC 18:2, LPC 18:1, LPC 16:0) and lysophosphatidylyl ethanolamine (LPE 18:1, LPE 20:4, LPE 18:2) in capons were higher than normal, indicating that castration enhanced lipid metabolism [16]. Furthermore, the NEFA levels in the CAM group were significantly higher than in the COM group, suggesting that castration also promoted fat mobilization. Juiciness of meat is positively correlated with fat content, and different fat contents may lead to differences in taste [8, 17]. The enhanced lipid metabolism may be one of the reasons why castrated chicken has a more fragrant aroma.

The types and contents of flavor compounds play a crucial role in determining the taste of meat. The flavor profile of chicken meat is intricately linked to its content of volatile organic compounds (VOCs). Key volatile substances present in chicken meat include aldehydes, alcohols, and alkanes, with hexanal and 1-octen-3-ol being predominant among the VOCs [18]. Inosinic acid stands out as a primary contributor to the savory umami taste of meat and serves as a vital indicator for assessing this flavor dimension [19, 20]. Notably, capons exhibit reduced levels of flavor-enhancing metabolites like inosine-5’-monophosphate (IMP), DL-malic acid, and 2-hydroxycinnamic acid compared to their regular counterparts, which may make the umami taste of normal chickens superior to that of capons. Carboxylic acid and derivative classes were identified, with metabolites such as 3-methylhistidine, 2-furoylglycine, L-histidine, creatinine, l-phenylalanine, creatine, and betaine found at significantly lower levels in capons compared to normal chickens. The metabolites play a role in the biosynthesis of amino acids. Additionally, metabolites including docosahexaenoic acid, cis-5,8,11,14, 17-eicosapentaenoic acid, arachidonic acid, adrenic acid, 9-oxo-ode, and palmitoleic acid metabolites which belong to the fatty acyl class, were observed at notably higher concentrations in capons chickens. These metabolites were involved in the biosynthesis of fatty acids. The distinct profiles observed in capons might indicate variations in their connective and adipose tissues relative to those of standard chickens.

To explore the mechanism behind differences in muscle quality caused by castration, transcriptome and proteome analyses were performed on capon chicken breast muscle. Bioinformatics analysis was conducted to identify key genes regulated by castration. The metabolic pathways were enriched through transcriptome, proteome and metabolome analyses. The regulatory network included the metabolites L-tryptophan, L-phenylalanine, L-histidine, nicotinamide, citric acid, benzoic acid, arachidonic acid, D-xylulose 5-phosphate, palmitic acid, creatine, isocitric acid, carnosine, histamine, betaine, creatinine, pantothenic acid, and l-anserine, the genes *GLUL*,* ACACB*,* AGPAT2*,* and HOGA1*, and the protein GSTA2.

GLUL is a key gene for inosine monophosphate synthesis [20]; its expression is positively correlated with IMP [21]. This result was further confirmed in this study. Both GLUL expression and inosine-5’-monophosphate (IMP) expression in capons were lower than those in normal chickens, indicating that the umami taste of capons may not be superior to that of normal chickens. ACACB is an important regulatory gene of lipid metabolism [22]; it is involved in fatty acid biosynthesis, the AMPK signaling pathway, and the insulin signaling pathway and is essential for adipogenesis, affecting IMF deposition [23] and abdominal fat traits in chickens [24]. The low expression of ACACB in capons alleviates its negative regulation of fatty acid metabolism, which is one of the reasons for the higher lipid content in capons than in normal chickens. AGPAT2 is a central gene of the triglyceride metabolism pathway [25], and its expression is regulated by estrogen [26]. After castration, AGPAT2 expression was low and was enriched in glycerolipid metabolism, fat metabolism, and absorption pathways. It also regulates lipid metabolism in capons. HOGA1 converts oxaloacetate to pyruvate and carbon dioxide [27], and its mitochondrial localization is related to the Krebs cycle and vital to adipose tissue [28]. HOGA1 was highly expressed in capons and involved in glyoxylate and dicarboxylate metabolism, arginine and proline metabolism, and other pathways and may promote adipogenesis, which is consistent with previous results [29]. GSTA2 is related to oxidative metabolism [30]. Glutathione metabolism eventually affects the formation of L-glutamate by regulating R-S-glutathione. The expression of GSTA2 was high in capons, but L-glutathione (reduced) was lower than that in controls, indicating that the antioxidant capacity of muscle was reduced after castration.

## Conclusion

*GLUL*,* ACACB*,* AGPAT2*,* HOGA1* and GSTA2 regulate the expression of L-tryptophan, L-phenylalanine, L-histidine, nicotinamide, citric acid, benzoic acid, arachidonic acid, D-xylulose 5-phosphate, palmitic acid, creatine, isocitric acid, carnosine, histamine, betaine, creatinine, pantothenic acid, and L-anserine, which may be the key mechanism for the difference in muscle quality of capons and normal chickens.

## Materials and methods

### Animals

Qingyuan Partridge chickens is a famous local chicken breed in China. A total of 40 healthy Qingyuan Partridge roosters at 64 days (the growth inflection point of Qingyuan partridge chickens), were selected and randomly divided into capon group (CA, 4 repetitions, each with 5 items) and control group (CO, 4 repetitions, each with 5 items). All chickens were anesthetized with a 2 mL injection of Procaine Hydrochloride (drug approval number: veterinary drug 070011674, Huamu, Jilin, China) as per the research of Masato Uehara [31]. The CA group underwent surgical castration to remove the testes, while the CO group received sham surgery at the same site. All chickens were raised at Guangdong Tinoo’s Food Group Co., Ltd. (Guangdong, China), following the feeding procedures for Qingyuan Partridge chickens. These roosters randomly were then slaughtered at 94 and 120 days (the listing age of Qingyuan partridge chickens), respectively (2 random items from each repetitions). Paraffin sections of breast muscle from the CAM and COM were collected and fixed in 10% neutral formalin. Breast muscle samples for transcriptome, proteomics, and metabolomics sequencing were collected and frozen in liquid nitrogen, and stored in a -80 °C. Sequencing was performed by Gene Denovo Biotechnology Co., Ltd. in Guangdong, China. The remaining chickens were euthanized by decapitation according to the American Veterinary Medical Association (AVMA) Guidelines for the Euthanasia of Animals (2020).

### Preparation of paraffin sections of muscle tissue

The breast muscle samples at 94 and 120 days from the CAM and COM groups (*n* = 3, respectively) were placed in a dehydration box with water and allowed to stand overnight. The tissues were then immersed in 75% ethanol for 4 h, 85% ethanol for 2 h, 90% ethanol for 2 h, and 95% ethanol for 1 h, and then in absolute ethanol and xylene for 1 h, and then finally embedded in wax for 2 h. The tissues were quickly picked up with preheated tweezers and placed into the embedding frame for fixation. The wax blocks took 30 min to solidify and were stored in the refrigerator at -20 °C after solidification. The wax blocks were sectioned to a thickness of 7 μm, and the tissues were flattened in a water bath at 43 °C. The wax blocks were baked in an oven at 45 °C for 12 h. The sections were placed in xylene and removed after 20 min in an incubator at 37 °C. Then, the sections were soaked in absolute ethanol for 20 min. Next, they were immersed in 95%, 90%, 80%, and 70% ethanol for 5 min each and washed for 2 min. The sections were stained with hematoxylin for 5 min, washed for 10 min, differentiated with 1% alcohol hydrochloric acid for 10 s, washed for 2 min, and observed under a microscope. The slide was stained with eosin for 3 min and washed for 5 min. After being dehydrated and sealed, the slices were placed under multiple microscopes (200 X) to observe the muscle fiber structure and different parts were took photos randomly. The fiber density and fiber size were analyzed by Image J software (version 1.8.0) [32]. SPSS 26.0 was used to perform one-way analysis of variance (ANOVA) statistical analysis of the results, and the *P* < 0.05 was considered significantly different [33]. Figures were drafted by GraphPad Prism 8 and presented as mean ± standard error of the mean (SEM) [34].

### Composition detected of breast muscle

Equal amounts and weights of breast muscle tissue was homogenized and evenly distributed on the test plate (*n* = 3, respectively). The analysis was conducted using near-infrared full-beam transmission technology. A meat composition analyzer (FOODSCAN2, Foss, Denmark) was used to determine the fat, moisture, salt, protein, collagen, and CIELAB color space parameters (L*, a* and b*) of the breast muscle [35], following the specified procedure.

### Shear force of breast muscle

Breast muscle samples measuring at least 6 cm × 3 cm × 3 cm (length × width × height) were collected as whole pieces (*n* = 3, respectively). The surface tendons, membranes, and fat were carefully removed. The samples were then heated in a constant-temperature water bath at 80 °C for 10 min using a 1500 W water bath system, after which they were cooled to room temperature. The shear force values were measured along the muscle fibers using a muscle tenderness tester (Tenovo, Beijing). Sampling locations were selected at least 5 mm from the sample edges, and the distance between the edges of two sampling points was no less than 5 mm. Each sample was measured three times.

### Non-esterified fatty acid assay

The NEFA was detected (*n* = 5, respectively) using the NEFA kit (A042-2, NJJCBIO, China). Appropriate tissue samples were weighed and homogenized in a 1:9 weight (g) to volume (ml) ratio with normal saline, resulting in a 10% homogenate. The homogenization process was performed mechanically under ice-water bath conditions. The homogenate was then centrifuged at 2500 RPM for 10 min. Afterwards, 4 µL of the supernatant was mixed with 200 µL of reagent A and incubated at 37 ℃ for 5 min. The absorbance value (A1) was measured using an Epoch microplate spectrophotometer (BioTek, China). Following this, 50 µL of reagent B was added to the mixture, which was then incubated again at 37 ℃ for 5 min before measuring the absorbance value (A2). Calculations were performed using a two-point calibration method. [36].

$$\eqalign{{C_{{\rm{NEFA}}}}_{\left( {{\rm{mmol/mg}}} \right)} & {{\Delta {A_{{\rm{sample}}}} - \Delta {A_{{\rm{blank}}}}} \over {\Delta {A_{{\rm{standard}}}} - \Delta {A_{{\rm{blank}}}}}} \times Cs{t_{\left( {{\rm{mmol/L}}} \right)}} \cr & \div {C_{{\rm{sample}}\left( {{\rm{g/L}}} \right)}} \times 1000 \cr}$$

### RNA sequencing

Total RNA of the breast muscle at 120 days in both CAM and COM groups (*n* = 3, respectively) was extracted according to the TRIzol reagent manufacturer’s instructions (TAKARA, Japan). Library construction and RNA-seq sequencing of muscle tissue were carried out. After separating the mRNA, it was fragmented by ultrasound, The fragmented mRNA was then reverse transcribed into cDNA. Following purification, the ends of the double-stranded cDNA were repaired, and the library was obtained by AMPure XP after an A-tail and a link-sequencing connector were added. Clean reads were generated using Fastp and aligned to the chicken ribosome database with Bowtie2 for subsequent transcriptome analysis. HISAT2 software was employed for alignment and analysis based on the reference genome. The expression levels and abundance distributions of all genes in each sample were calculated using StringTie. Differential gene expression analysis was performed using DESeq2. Genes with an adjusted p-value (FDR) < 0.05 and an absolute log2 fold change (|log2FC|) > 1 were considered significantly differentially expressed. Enrichment analyses of GO terms and KEGG pathways were conducted.

### Real-time quantitative fluorescence

After homogenizing an appropriate quantity of breast muscle tissue at 120 days (*n* = 3, respectively), we extracted the total RNA following the reagent protocol of kit (R401, Vazyme, China) and assessed the RNA integrity via gel electrophoresis. The cDNA was synthesised using the RNA reverse transcription kit (R323, Vazyme, China). Subsequently, performed PCR according to the guidelines provided in the real-time fluorescent quantitative PCR kit (Q711, Vazyme, China). The total volume of the PCR reaction was 20 µL, consisting of 10 µL of 2× ChamQ Universal SYBR qPCR Master Mix, 0.4 µL of each forward and reverse primer, 1 µL of DNA template, and the remaining volume made up with ddH_2_O. The real-time fluorescent quantitative PCR protocol was included an initial denaturation step at 95 °C for 30 s, followed by 40 cycles of denaturation at 95 °C for 10 s and annealing at 60 °C for 30 s, the final steps consist of 95 °C for 15 s, 60 °C for 60 s, and 95 °C for 15 s by QuantStudio5 (Applied Biosystems, America). Primer sequences were designed using Primer Premier 5.0 software, based on the mRNA sequences of the target gene *GSTA2 (NM_001001776.2)*,* ACACB (XM_040648481.2)*,* HOGA1(XM_001233351.7). GLUL (NM_205493.2)*,* AGPAT2 (XM_001235299.7)* and the reference gene *GADPH (NM_204305.2)* from the NCBI database. Details of the primers were shown in Table 6. The primers were synthesized by Sangon Biotech Co., Ltd (Shanghai, China).

**Table 6:** The primers of genes for RT-PCR

Prime Name	Primer sequence	Annealing temperature
GLUL-F	CATCTGGATCGACGGGACTG	60
GLUL-R	TGCAGACTGGCGGTTGTATT	60
ACACB-F	TCTGTGGTGCTCTGAACGTC	60
ACACB-R	CCATCGTAGGAGAGCAGCAG	60
AGPAT2-F	GGCTCATCACATACCTCGGG	60
AGPAT2-R	CCTGGACAGCGAGGTGAAAT	60
HOGA1-F	GGTCGTGACACCCTGCTATT	60
HOGA1-R	CGTCTTGTGGACAATCAGCC	60
GSTA2-F	GCTGGGGTTGAATTCGAGGA	60
GSTA2-R	ATCGGCTGCTTGAAAAGGGA	60

### DIA procedures

Breast muscle samples at 120 days from the CAM and COM groups (*n* = 3, respectively) were vortexed and lysed for 30 min on ice with lysis buffer (1% SDS, 8 M urea, and 1 mg/ml protease inhibitor cocktail). Then, the samples were homogenized for 2 min and centrifuged at 12,000× g for 15 min at 4 ℃. The supernatant was collected and quantified by Pierce™ Quantitative Colorimetric Peptide Assay (23275, Thermo Fisher Scientific, MA, USA). The mixture was then labeled using the iTRAQ-8Plex Isobaric Mass Tag Labeling Kit (Thermo Fisher Scientific, MA, USA), redissolved, and fractionated by high pH separation using an Ultimate 3000 system (ThermoFisher Scientific, MA, USA) in 0.1% formic acid in water. The peptides were analyzed via online nanospray LC‒MS/MS on an Orbitrap Fusion™ Lumos™ Tribrid™ coupled to an EASY-nLC 1200 system (Thermo Fisher Scientific, MA, USA). The mass spectrometer operated in data-dependent acquisition mode. Tandem mass spectra were processed by PEAKS Studio version X+ (Bioinformatics Solutions Inc., Waterloo, Canada) [37]. Peptides were filtered to include at least one unique form. Protein abundance was analyzed using Student’s t-test. DEPs were filtered based on a fold change > 1.2 and the presence of at least 1 unique peptide with *P* < 0.05. GO and KEGG enrichment analyses were also conducted [38].

### Metabolome sequencing

Breast muscle samples at 120 days from the CAM and COM groups (*n* = 5, respectively) homogenate (100 mg). The samples were then resuspended with 80% methanol and vortexed. After incubation on ice for 5 min, the mixture was centrifuged at 15,000× g for 20 min at 4 °C. The supernatant was diluted with 53% methanol, centrifuged at 15,000× g for 20 min at 4 °C, and then injected into the LC‒MS/MS system [39]. UHPLC‒MS/MS analyses were conducted by Gene Denovo Co., Ltd. (Guangzhou, China). Compound Discoverer 3.1 (CD3.1, Thermo Fisher) was employed to process the raw data for peak alignment, peak picking, and quantitation for each metabolite. Peak intensities were normalized and used to predict molecular formulas. The peaks were matched with the mzCloud (https://www.mzcloud.org/), mz Vault and mass list databases [40]. Multivariate statistical analysis was combined with variable importance in projection (VIP) values from OPLS-DA and t-tests to identity differential metabolites [41–43]. The threshold for significance was set as VIP ≥ 1 and *P* < 0.05. R (version R-3.4.3), Python (Python 2.7.6) and CentOS (release 6.6) software were used for statistical analyses [40].

### Integrative analysis

A pathway function model, a bidirectional O2PLS model, and a correlation coefficient model were analyzed based on the two sets of data for gene or protein expression and metabolite abundance using R software (version R-3.4.3) [44]. Transcriptome and proteome association number analysis, four-quadrant diagrams, and GO/KO association analysis were conducted based on the expression levels and functional information of genes and proteins using R software (version R-3.4.3) [45].

## Data Availability

The transcriptome data that support the findings of this study have been deposited into the CNGB Sequence Archive (CNSA) of the China National GeneBank DataBase (CNGBdb) with accession number CNP0004287. The proteomic datasets generated and analyzed during the current study are available in the Center for Computational Mass Spectrometry database with accession number MSV000091807.
